# Long-term survival of a recurrent gallbladder carcinoma patient with lymph node and peritoneal metastases after multidisciplinary treatments: a case report

**DOI:** 10.1186/s40792-016-0135-8

**Published:** 2016-02-11

**Authors:** Koichi Tomita, Kiminori Takano, Motohide Shimazu, Masaaki Okihara, Toru Sano, Naokazu Chiba, Shigeyuki Kawachi

**Affiliations:** Department of Digestive and Transplantation Surgery, Tokyo Medical University Hachioji Medical Center, 1163 Tatemachi, Hachiojishi, Tokyo 193-0998 Japan

**Keywords:** Gallbladder carcinoma, Peritoneal metastasis, Long-term survival, Multidisciplinary treatment, Resection, Chemoradiotherapy

## Abstract

**Background:**

Gallbladder carcinoma with peritoneal metastasis has a poor prognosis, with a median survival time of 4.8 months. We report the survival of a patient with gallbladder carcinoma with peritoneal metastasis for 7.6 months owing to treatment with tumor resection after chemoradiotherapy.

**Case presentation:**

A 69-year-old man was referred to our hospital for gallbladder carcinoma with hepatic invasion. Cholecystectomy was performed along with S4a and S5 hepatectomy and extrahepatic bile duct resection with lymph node dissection. The postoperative pathological diagnosis was moderately differentiated adenocarcinoma, T3, N0, M0, stage IIIA by the International Union Against Cancer TNM classification. Despite treatment with gemcitabine, the common hepatic artery and para-aortic lymph nodes showed metastases after 3 months from surgery. Although a combination of cisplatin, gemcitabine, and radiotherapy reduced the size of the lymph node metastasis, the peritoneal metastasis persisted. The peritoneal metastasis responded to chemoradiotherapy using tegafur-uracil and leucovorin, but it recurred. The metastasis was resected after 3 years and 9 months from the first surgery, and chemotherapy was discontinued. Seven years and 6 months after the initial surgery, the patient exhibited no signs of tumor recurrence or metastasis.

**Conclusions:**

Multidisciplinary treatment including resection without residual tumors could achieve complete remission of gallbladder carcinoma with lymph node and peritoneal metastases in the selected patient.

## Background

Gallbladder carcinoma (GBC) is a fatal disease with a poor prognosis, owing to the tendency of the tumor to metastasize early to the regional lymph nodes and spread into the liver bed [1]. GBC with peritoneal metastasis has an exceptionally poor prognosis, with a median survival time (MST) of 4.8 months [2].

Prognostic factors for GBC invasion include liver metastasis, perineural invasion [3], lymphatic invasion, and lymph node metastasis [4]. Factors associated with survival after GBC resection include lymph node dissection [5] with extrahepatic bile duct resection [6] and resection of the hepatic bed [7] as well as S4a and S5 hepatectomy [8]. According to these reports, there is no doubt that resection without residual tumors and lymph node dissection or hepatic resection are important for achieving complete remission.

However, GBC with distant metastases to the liver, the lymph nodes beyond the hepatoduodenal ligament, and the peritoneum are typically thought to be contraindications for surgery and are treated best by chemotherapy and radiotherapy, although the MST of patients with such disease is less than 1 year [2]. We report the survival of a patient with GBC with lymph node and peritoneal metastasis for 7.6 months; this was achieved by controlling disease progression with chemoradiotherapy and resection of the peritoneal metastasis.

## Case presentation

A 69-year-old man was diagnosed with GBC with hepatic invasion after a 2-year follow-up for right breast cancer surgery, during which his serum carcinoembryonic antigen (CEA) level was elevated. He was referred to our hospital in Jun. 2007.

**Fig. 1:**
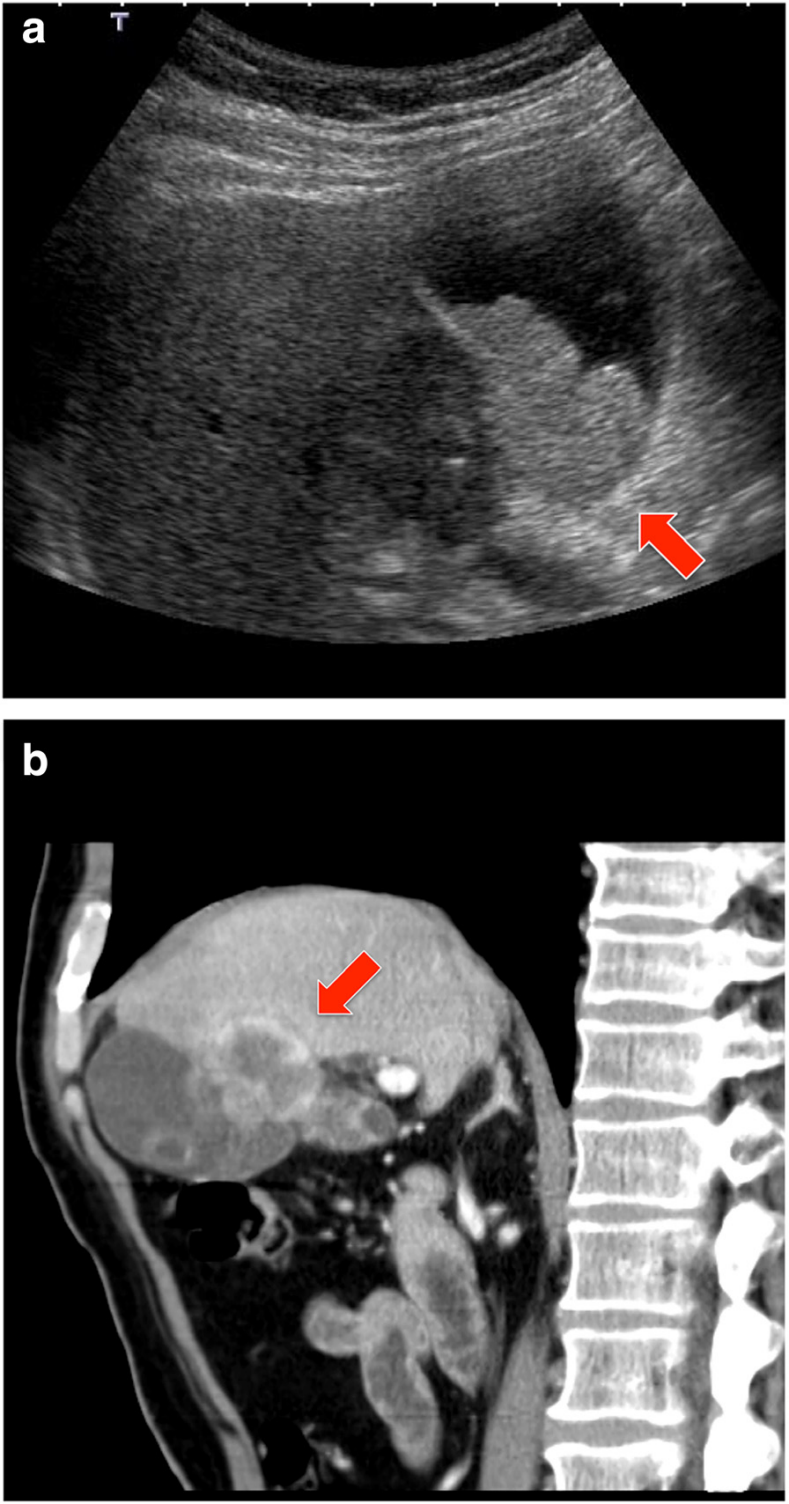
An ultrasonography image (**a**) and a sagittal section of a computed tomography scan (**b**) showing the gallbladder carcinoma. The *red arrows* indicate the tumor mass at the neck of the gallbladder, 37 × 30 mm in diameter, involving the liver bed

Preoperatively, abdominal ultrasonography and computed tomography (CT) scan revealed a tumor (37 × 30 mm in diameter), involving the liver bed from the neck of the gallbladder (Fig. 1). Endoscopic ultrasonography did not reveal any cystic duct invasion; however, metastasis to the lymph node near the common bile duct was suspected on CT scan. Distant metastasis, including to the liver, was not observed. The results of a blood test on admission indicated that liver and kidney functions were normal. The levels of tumor markers were as follows: CEA, 10.3 ng/mL and carbohydrate antigen (CA) 19-9, 785.24 U/mL.

Cholecystectomy with hepatectomy of S4a and S5, lymph node dissection of the hepatoduodenal ligament, resection of the extrahepatic bile duct, and Roux-en-Y choledochojejunostomy were performed in Jul. 2007. R0 resection was accomplished. The postoperative course was uneventful.

**Fig. 2:**
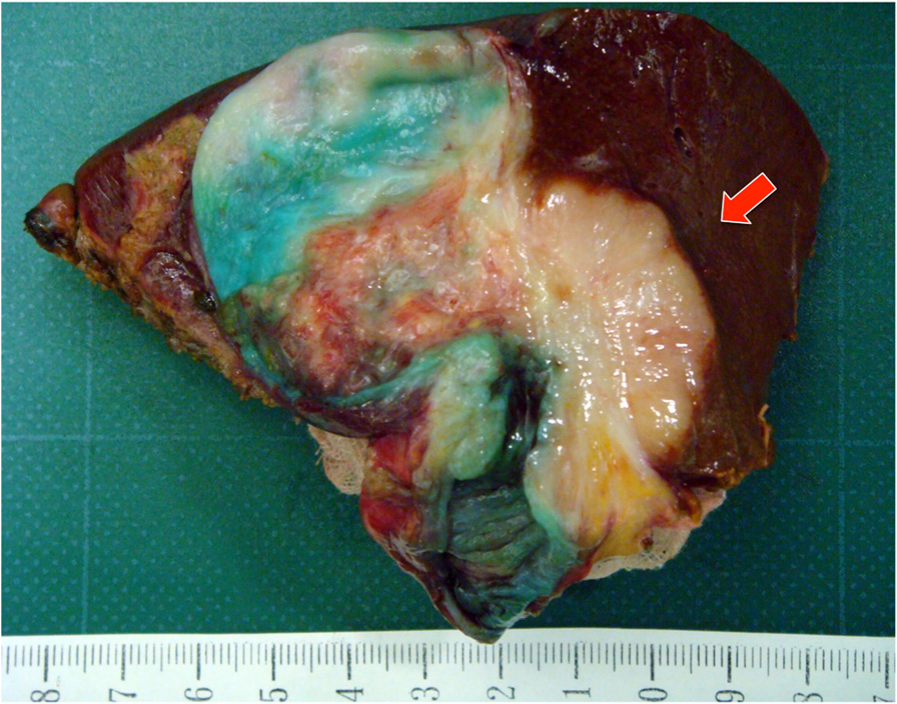
The cut surface of the resected specimen. The *red arrow* indicates the tumor mass at the neck of the gallbladder involving the liver bed, which was pathologically diagnosed as a moderately differentiated adenocarcinoma. The lymph node, cystic duct, cystic artery, and other surrounding organs were negative for invasion. Hepatic metastasis was not observed. The gallbladder was injected with indocyanine green, which was used to observe the cystic vein perfusion area

According to the seventh edition of the Tumor Nodes and Metastasis (TNM) Classification of the International Union Against Cancer, the postoperative pathological diagnosis was moderately differentiated adenocarcinoma of the gallbladder, T3, N0, M0, stage IIIA (Fig. 2).

**Fig. 3:**
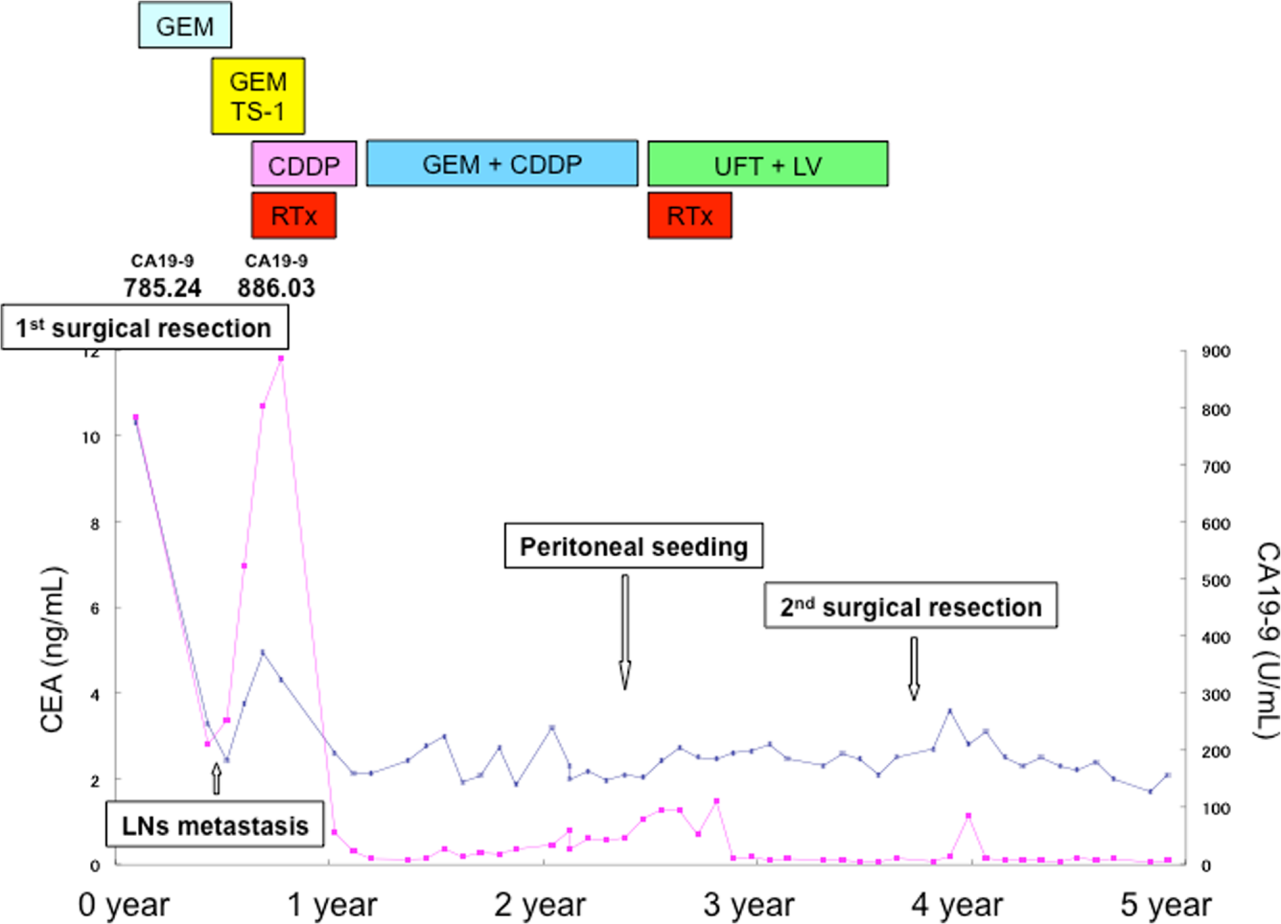
Clinical course and tumor marker levels. The *blue line* illustrates the level of carcinoembryonic antigen (*CEA*), and the *pink line* illustrates the level of carbohydrate antigen (*CA*) 19-9. Abbreviations: *GEM* gemcitabine, *TS-1* combination capsules of tegafur, gimeracil, and oteracil potassium, *CDDP* cisplatin, *RTx* radiotherapy, *UFT* uracil and tegafur, *LV* leucovorin, *LNs* lymph nodes

**Fig. 4:**
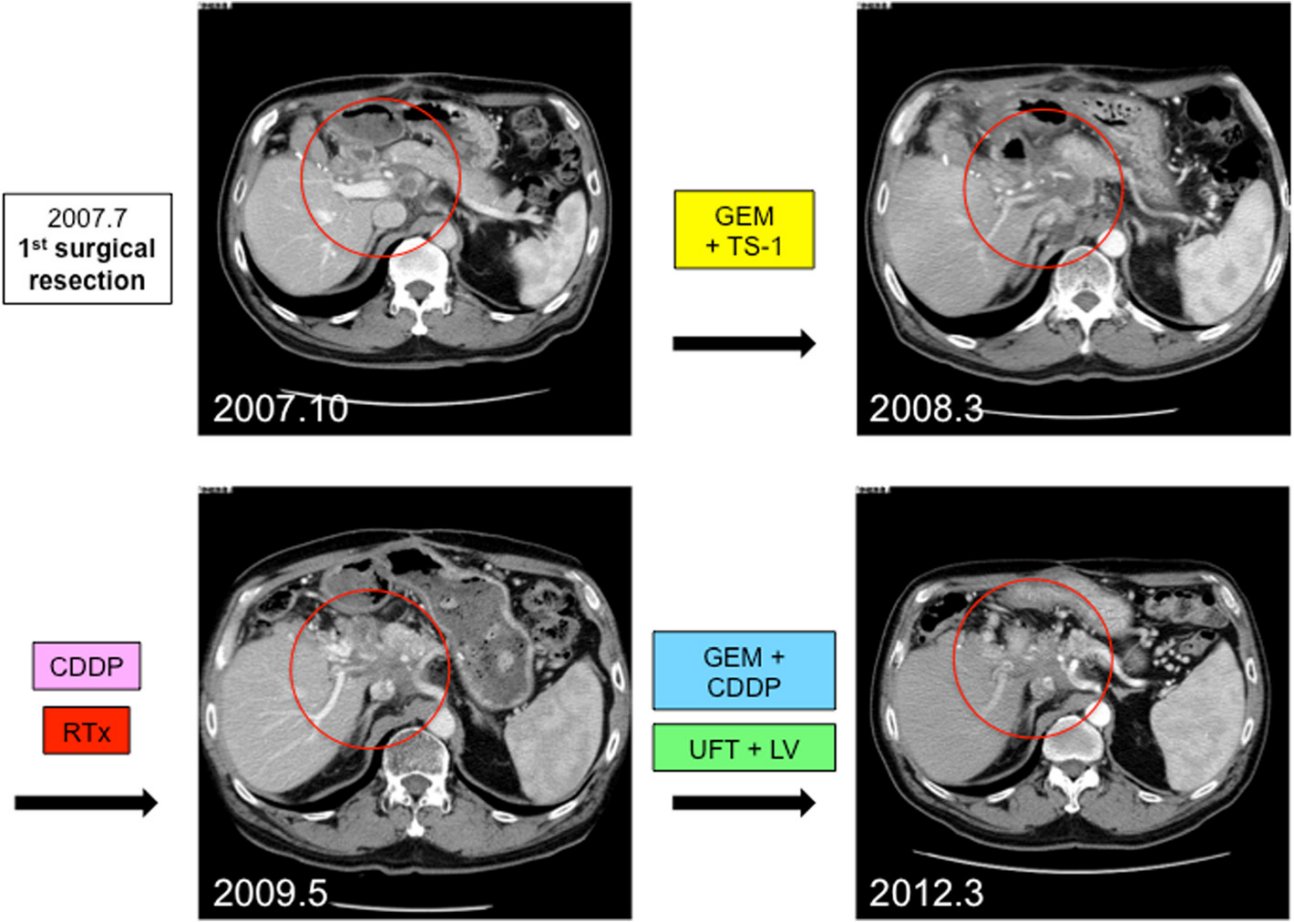
Progression of the metastasis to the common hepatic artery and para-aortic lymph nodes. The lymph node metastasis was detected 3 months after the first resection (**a**). Despite administration of gemcitabine (*GEM*) plus TS-1 and cisplatin (*CDDP*) plus radiation, the metastasis enlarged and involved the portal vein (**b**). However, the growth rate remarkably decreased after GEM plus CDDP (**c**) and uracil and tegafur plus leucovorin (**d**) administration and has maintained the same size

The patient’s clinical course and associated changes in tumor markers are illustrated in Fig. 3. Adjuvant chemotherapy was administered (gemcitabine [GEM], 1000 mg/m^2^, biweekly). After 8 cycles of chemotherapy, the patient’s CA 19-9 level had increased to 210.51 U/mL and metastases to the common hepatic artery lymph nodes and para-aortic lymph nodes were detected in Oct. 2007 (3 months post-surgery) on CT scan. Therefore, the chemotherapy regimen was changed to GEM (1000 mg/m^2^, days 1 and 8) and TS-1 (a combination capsules of tegafur, gimeracil, and oteracil potassium, 60 mg/m^2^, daily, 2 weeks on/1 week off). GEM and TS-1 were discontinued when the patient developed thrombopenia accompanied by elevation of CA 19-9 to 801 U/mL in Jan. 2008. A CT scan revealed that the common hepatic artery lymph node and para-aortic lymph node metastases had increased in size by surrounding the common and proper hepatic arteries; the metastases reached the portal vein bifurcation (Fig. 4).

In Mar. 2008 (8 months post-surgery), a CT scan revealed an enlargement of the lymph node metastasis with invasion into the portal vein, celiac artery, superior mesenteric artery, duodenum, pancreas, and inferior vena cava. A combination of low-dose cisplatin (CDDP, 6 mg/body, daily) and X-ray radiation consisting of 50 Gy/25 fr was administered. After chemotherapy and radiation were completed in Jun. 2008 (11 months post-surgery), CA 19-9 levels had decreased to 22.53 U/mL.

After the decrease in CA 19-9 levels, GEM (1000 mg/m^2^) and CDDP (15 mg/m^2^) were re-administered on days 1 and 8 (2 weeks on/1 week off). CA 19-9 decreased to normal levels after three courses of treatment. GEM and CDDP were continued, and stable disease was maintained according to the Response Evaluation Criteria in Solid Tumor (RECIST). In Dec. 2009 (2 years and 5 months post-surgery), after 28 courses of treatment had been administered in total, a new metastatic lesion was detected near the colon of the hepatic flexure on a CT scan and was diagnosed as a peritoneal metastasis.

**Fig. 5:**
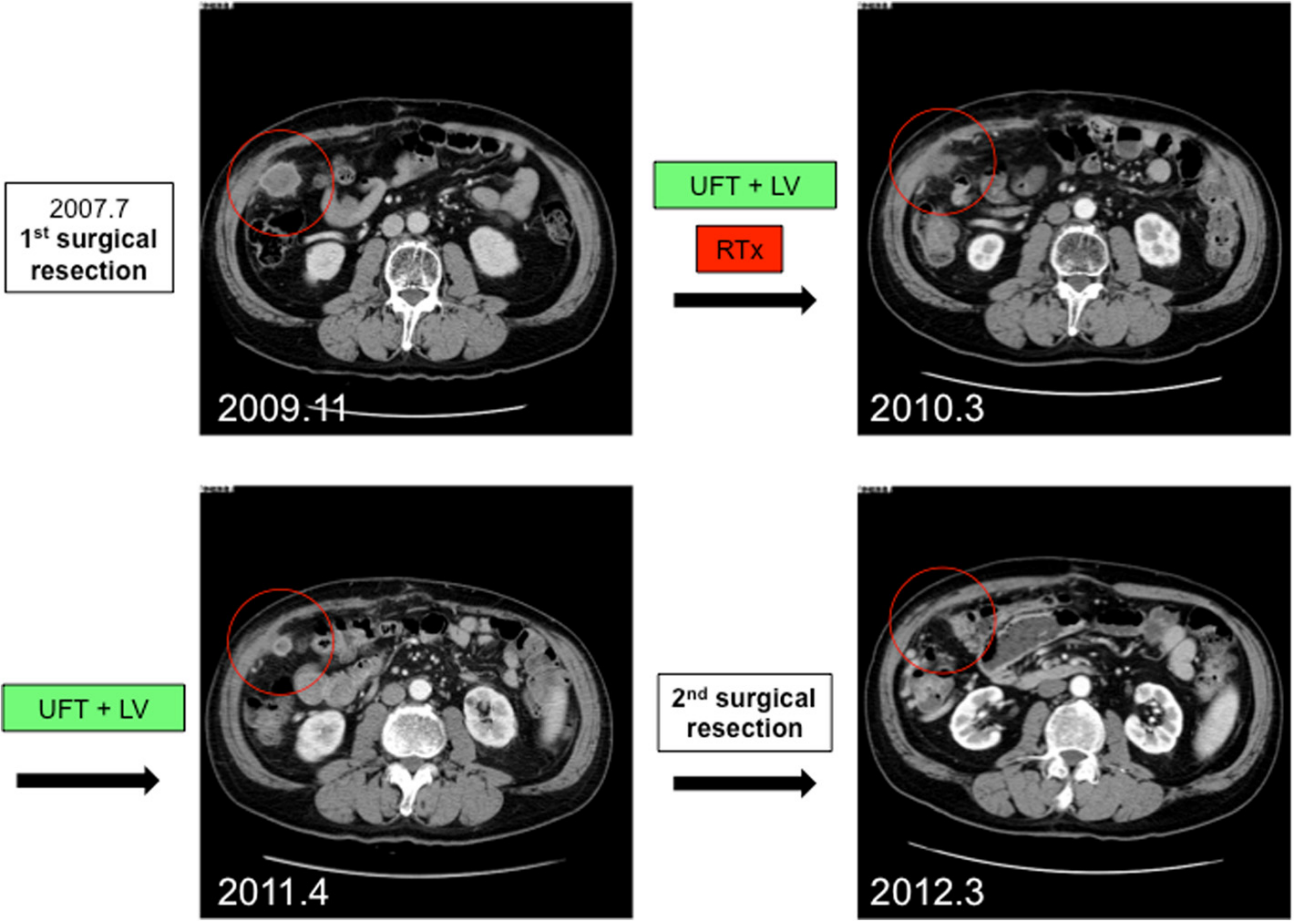
Change in peritoneal metastasis near the hepatic flexure. Peritoneal metastasis was detected 2 years after the first resection (**a**). It decreased in size after treatment with gemcitabine plus cisplatin and uracil and tegafur (*UFT*) plus leucovorin (*LV*) with radiation (**b**). However, the peritoneal metastasis began to enlarge during UFT plus LV administration (**c**) and was resected in a second surgery in Apr. 2011 (**d**)

Owing to the discovery of the additional metastasis, the chemotherapy regimen was changed to uracil and tegafur (UFT, 300 mg/body) and leucovorin (25 mg/body), which were administered daily (4 weeks on/1 week off), in Jan. 2010 (2 years and 6 months post-surgery). X-ray radiation of 40 Gy/20 fr was also administered. After treatment, the levels of tumor markers decreased to normal, and a CT scan revealed reduced tumor size of the peritoneal metastasis (Fig. 5).

In Apr. 2011 (3 years and 9 months post-surgery), after 16 courses of UFT and leucovorin treatment, the levels of tumor markers had once again increased and the size of the peritoneal metastasis had increased. Radiotherapy treatment was no longer possible because the patient had reached the maximum dose allowable and because of the risk for adverse gastrointestinal effects. Multiple imaging modalities including CT, magnetic resonance imaging (MRI), and positron emission tomography (PET/CT) revealed that the peritoneal metastasis was isolated. Also, the lymph node metastases of hepatoduodenal ligament and para-aorta maintained stable disease and did not show any viability. Therefore, the peritoneal metastasis was resected in Apr. 2011. Open laparotomy findings did not reveal any peritoneal dissemination except the isolated peritoneal metastasis. The metastatic mass was 18 × 15 mm in size and was resected without a residual tumor. The postoperative pathological diagnosis was a metastatic adenocarcinoma. The effect of radiotherapy was also observed, and its effect was evaluated as grade 1b (about half of tumor cells showed highly change).

After resection, chemotherapy was discontinued because the tumor marker levels were within normal limits. PET/CT scan obtained in Nov. 2011 did not reveal any tumors.

At the time of reporting, 7.6 months have passed since the primary tumor was removed, and 3 years and 9 months have passed since the peritoneal metastasis was resected. At this time, the levels of tumor markers are within normal limits and tumor recurrences have not been detected on CT scan.

## Discussion

In this report, we demonstrate a rare case of long-term disease-free survival in a patient with recurrent GBC with lymph node and peritoneal metastases by using multidisciplinary treatments including resection, chemotherapy, and radiotherapy.

The poor prognosis of advanced GBC, such as that with distant metastasis to the liver, peritoneum, or lymph nodes of the extrahepatoduodenal ligament or para-aorta, is well recognized. Because of the difficulty associated with complete resection, chemotherapy or radiotherapy is usually utilized in these advanced GBC cases [9]. The main chemotherapeutic agents for GBC include GEM, CDDP, and 5-fluorouracil, alone or in combination. According to a phase II study, 1000 mg/m^2^ of GEM elicited a 17.5 % response rate (RR) with an MST of 7.6 months in cases of advanced GBC [10]. TS-1 elicited a 35 % RR with an MST of 9.4 months [11]. The combination of GEM and CDDP elicited a 21 to 48 % RR with an MST of 4.6 to 11.0 months in several reports [12–15]. Although these were cohort studies, randomized trials evaluating the effect of these drugs alone and with other agents, such as epirubicin, etoposide, leucovorin, mitomycin C, and capecitabine, have been performed but have not demonstrated positive outcomes [16–18]. Adjuvant chemotherapy has also been thought to play a marginal role in the treatment of GBC.

Thus, resection without residual tumors, if possible, still has an important role in improving the prognosis of advanced GBC. Several previous reports support the notion that radical resection of advanced GBC enhances survival [19–21]. Watanabe et al. reported the survival of a patient with advanced GBC for more than 12 years after curative surgery that included cholecystectomy, liver bed resection, pancreatoduodenectomy, right hemicolectomy, and anterior abdominal wall resection [22].

Even in cases of metastatic GBC, if the metastatic lesions were surgically resectable and well controlled with chemotherapy and/or radiotherapy, resection with negative margins might result in long-term survival and complete remission. A previous report showed that patients with advanced GBC could benefit from resection even when para-aortic lymph node metastasis and/or liver metastasis are present [2]. Scaringi et al. reported a patient with recurrent GBC who was treated with iterative resection and was alive and disease-free 5 years after the final surgical procedure [23]. In their case, fluorouracil and leucovorin were administered as adjuvant chemotherapy; however, splenic metastasis appeared after 1 year from the first operation. Even though the splenic metastasis was resected, pancreatic and gastric metastases appeared after another year. Distal pancreatectomy en bloc with sleeve gastrectomy was performed, and the patient was alive for 5 years. Only a few reports have demonstrated the long-term survival of patients with recurrent GBC treated with resection of metastatic lesions, similar to our case.

Our current case demonstrated that long-term survival could be achieved with resection of metastatic lesions of GBC, if the metastatic lesion was isolated, resectable, and well controlled with chemoradiotherapy. The criteria to confirm the isolation of metastasis and the definition of “well controlled” are controversial, and multiple imaging modalities including CT, MRI, and PET/CT could be helpful to confirm it. Also, the definition of resectability is different between surgeons or institutions, and further investigations are needed to provide consistent evidence.

## Conclusions

In conclusion, despite significant developments in the fields of chemotherapy and/or radiotherapy, resection without residual tumor might have an important role in improving the prognosis of recurrent GBC. Resection should be considered if the metastatic lesion is isolated and well controlled with chemoradiotherapy in order to achieve complete remission and/or long-term survival.

## Consent

Written informed consent was obtained from the patient for publication of this case report and any accompanying images. A copy of the written consent is available for review by the Editor-in-Chief of this journal.
